# Progressive Medicine

**Published:** 1906-01

**Authors:** 


### BOOK REVIEWS.

---

PROGRESSIVE MEDICINE. Vol. IV, December, 1905. A Quarterly Digest of Advances, Discoveries and Improvements in the Medical and Surgical Sciences. Edited by Hobart Amory Hare, M.D., Professor of Therapeutics and Materia Medica in the Jefferson Medical College of Philadelphia. Octavo, 367 pages, 41 engravings, and 5 full-page colored plates. Per annum, in four cloth-bound volumes, $9.00; in paper binding, $6.00; carriage paid to any address. Lea Brothers & Co., Publishers, Philadelphia and New York.

The general excellence of the above work is in keeping with the former volumes and contains a surprisingly large amount of valuable information upon the recent literature and progress of medicine.

The diseases of the digestive tract and allied organs, pancreas, liver and peritoneum are gone over by J. Dutton Steele, while the genito-urinary diseases are ably discussed by William T. Belfield. John R. Bradford reviews the recent literature on kidney diseases. Anesthetics, fractures, dislocations, amputations, surgery of the extremities and orthopedics are thoroughly taken up by Joseph C. Bloodgood. The last 77 pages comprise a practical therapeutic referendum, in which various drugs are taken up in an alphabetical order and their important features, brought out by recent researches, are discussed.

